# Progress in Medicine

**Published:** 1897-08-07

**Authors:** 


### PROGRESS IN MEDICINE.

### DISEASES OF THE HEART AND CIRCULATION.

**Phonendoscope.**—It was contended by Bianchi, the inventor of this new instrument, which was to replace the old-fashioned stethoscope, that no vibrations were lost, whilst sounds were not exaggerated as by the microphone. However, Egger[1] says that a number of extra sounds are liable to be produced, as, for instance, by shaking the tubes. The heart sounds are certainly heard with greater loudness, and over a more extended area than with the ordinary stethoscope; but this, though useful for the deaf, is hardly so for the ordinary person. Thus, it is not desirable to hear the cardiac sounds whilst listening over the apices of the lungs. In regard to lung sounds, some overtones are conducted whilst others are not. Bronchial breathing and moist sounds are not intensified, rhonchi are intensified, whilst consonating râles are not. Thus, a metallic clang may be lost, whilst sounds may appear metallic which are not so. Egger, then, comes to the conclusion that the phonendoscope will not replace the stethoscope for ordinary ausculatory work, but has a limited field of usefulness in determining the existence of doubtful cardiac murmurs.

**Roentgen Rays.**—It has now become an accepted fact that the Roentgen rays will enable us to discover the position and size of the heart, and, to some extent, its movements, and thus furnish us with a valuable additional means of diagnosis in obscure cardiac conditions. This may be done either by a permanent photographic record on a plate or by a fluorescent screen. Campbell[2] says that the method by direct vision is preferable; a piece of paper may be fixed to the back of the fluorescent screen and a tracing made along the edge of the shadow thrown by the heart. Satterthwaite[3] confirms the majority of Campbell's conclusions, but says that Campbell's plan of putting the Crook's tube opposite the spine of the seventh dorsal vertebra on the left side, *i.e.*, opposite the centre of the heart, is apt to produce some distortion and enlargement of the shadow. He prefers to first ascertain the outline of the cardiac dulness as far as possible by percussion, and then to get a tracing whilst the light is moved opposite these points. The method is of particular importance in the examination of cases submitted to the Schott plan of treatment, in cases of emphysema where percussion gives no reliable results, and in doubtful cases of aneurism and mediastinal tumour.

**Malignant Endocarditis.**—In view of the favourable results which have in some cases attended the use of antistreptococcic serum in cases of malignant endocarditis, a paper of Phear[4] on the etiology and diagnosis of the conditions is of some interest. It must be clear, in the first place, that there is endocarditis, and secondly there must be evidence that the endocarditis is infective. Endocarditis is evidenced by signs and symptoms which are the mechanical outcome of the diseased state of the valve, and which are precisely similar to those of rheumatic or other origin. Some help may be afforded by the fact that malignant endocarditis may attack parts of the endocardium not commonly involved by simple endocarditis, *e.g.*, the right side of the heart; also by the development of fresh murmurs whilst the patient is under observation, though this of itself is not certainly indicative of advancing disease, for it may be due to either recuperation or failure in the heart's action. Evidence of the endocarditis being infective is found in the existence of fever and in the occurrence of embolism. In the absence of fever the diagnosis of infective endocarditis cannot reasonably be made. The fever, as is well known, is very variable in its character, and may be of pyæmic, typhoid, or even of malarial type. Instances of the last have recently been recorded by Dock[5]; as a rule, however, the fever can only be described as irregular. The occurrence of embolism is of great importance, especially if evidence is forthcoming that the embolus is septic; for instance, a sudden rigor, followed by swelling and tenderness of the spleen, is valuable corroborative evidence. Thus, the association of valvular disease, recurring embolism, and fever for which no adequate cause can be found, is essential for a positive diagnosis of infective endocarditis, though in many other cases a suspicion as to its nature may be well founded. The micro-organisms most commonly found are the staphylococcus pyogenes aureus, streptococcus pyogenes, and the diplococcus pneumoniæ. *A priori* it appears probable that the injection of antistreptococcic serum would do good only in those cases due to the streptococcus pyogenes, and would have no beneficial result in those due to other micro-organisms; and streptococcic cases have been recorded, *e.g.*, by Sainsbury,[6] in which recovery has resulted from the use of this serum, though failure has resulted, as in the case reported by Fox.[7]

What is wanted is a means of ascertaining whether streptococci are the cause of any given case of infective endocarditis. In some cases, *e.g.*, Sainsbury's, these have been found in the blood during life; but this is not always possible; none were discovered in the blood in Fox's case, though their existence was confirmed at the post-mortem examination. Cases have been recorded by Thayer and Blumer[8] in which the gonococcus was the cause of the disease; in these the use of the antistreptococcic serum would effect no good; but obviously it should be tried in every case in which the disease is not certainly due to organisms other than streptococci. The doses hitherto used have been 20 and 10 c.c. repeatedly injected.

**Syphilitic Disease of the Heart Wall.**—We owe to Phillips[9] a valuable paper on the pathology, diagnosis, and treatment of syphilitic lesions of the cardiac wall. Syphilis may produce the same changes in the heart as it does in other internal viscera—either a general fibroid degeneration or gummata, or both of these results. In the heart the change is a replacement of the muscular tissue by a round-celled fibroid tissue, which, whether it occurs as nodules or as a more widely-spread change, leads to dilatation or hypertrophy, or mere rigidity of the heart wall, and has this uniform result that it impairs or interferes with the contraction of the heart. Pathologically, gummata are characteristic, whilst diffuse fibroid change may be due to causes other than syphilis, so that it is not possible to be sure of the etiology, except by the history or by the joint occurrence of gummata. Very small gummata may exist in the cardiac wall without giving any recognisable evidence of their presence during life, and, indeed, without producing any symptoms whatever. But gummata of larger size are of great importance, for they may cause sudden death in the prime of life in otherwise healthy persons. Necropsies show that it is only when a gumma is in the wall of the left ventricle and near the apex that it is capable of producing sudden death. But the histories of the cases, and such clinical evidence of those cases which have cleared up under treatment, show that there have generally been symptoms which, if recognised, might have led to successful treatment. These symptoms appear to be one or more of the following: Palpitation, subjective sensations about the precordium, attacks of angina pectoris, giddiness, faintness, or actual syncopal attacks, epileptiform seizures, and alterations in the force and frequency of the pulse, especially great rapidity, or in some cases irregularity. Two of these, angina and tachycardia, merit special attention. Angina is usually associated with physical signs or symptoms of heart or valvular disease, which can leave no doubt that the angina is dependent on the heart's condition. A gumma of the heart wall may produce angina with scarcely any other symptoms or signs of heart disease—a characteristic it shares with fatty degeneration of the heart, aneurism of the very root of the aorta within the pericardial sac, and also aneurism of the cardiac wall. Fatty degeneration, however, as a rule, occurs after fifty years of age, whereas syphilitic disease is set up generally at an earlier period, and a fatty heart is usually associated with other evidences of its presence, whilst in the case of gummata there will be a history or evidence of syphilis. In angina due to aneurism the sense of impending death is not so marked a feature, the pain often radiates down both arms, and there is a ringing second sound and frequently a diastolic murmur. In cases, then, of angina occurring before, say fifty years of age, unaccompanied by any other evidences of heart disease, a presumption of a gumma as the cause exists, and this may be strongly increased if there is a history of syphilitic infection. Tachycardia may be one of the earliest signs of syphilitic disease of the heart wall, but if unaccompanied by other symptoms, it must be very difficult, if not impossible, to distinguish it from a mere functional state.

There is no recorded case of sudden or gradual death from gumma of the right ventricle; but in all the cases of extensive fibroid disease of the right ventricle, dyspnœa has been one of the earliest and most constant symptoms. So that if dyspnœa occurs without lung disease, or left-sided heart disease, or other obvious cause, it is well to remember its possible dependence on some fibroid change in the wall of the left ventricle, and a syphilitic history may confirm such a view. Cases of general fibroid disease of the heart may be divided into two classes—those in which the weakened muscular tissue yields to the intracardiac pressure, and dilatation results; and cases in which the heart-wall becomes toughened, stiffened, and thinned, with much loss of power but without dilatation. When the left ventricle dilates the evidences are the same as those of dilatation from any other cause, *i.e.*, subjective sensations about the cardiac region, or even angina pectoris, giddiness, faintness, or actual syncopal attacks with alterations in the force or frequency of the pulse, together with the physical signs of dilatation of the left ventricle. The symptoms are often more marked than the amount of dilatation present would lead one to expect, probably because the fibroid change resists the dilatation to some extent, though greatly enfeebling the heart power. Death may occur suddenly or only after dilatation of the right ventricle, with symptoms of tricuspid regurgitation. When the right ventricle becomes affected, suffocative attacks of dyspnœa, ascites, albuminuria, with physical signs of tricuspid regurgitation result, but sudden death in the midst of apparent health apparently does not occur. Diffuse fibroid change without dilatation may occur in the case of the left ventricle; a progressive loss of power ensues, and the action of the heart may get extraordinarily weak, the apex beat becoming imperceptible and the first sound almost or quite inaudible, and there is a tendency to giddiness, faintness, and syncopal attacks. Such cases are gradual in development, and are often put down to "fatty" or "weak" heart. Fibroid change of the right ventricle without dilatation produces dyspnœa as its chief symptom, as in cases where there is dilatation. The heart may in some cases appear hypertrophied, due to the increase of fibroid not of muscular tissue. The diagnosis rests on the evidence of hypertrophy without obvious cause, without valve disease, albuminuria or history of pericarditis, or severe muscular exertion. The diagnosis is supported where the increased bulk of the heart, as estimated by physical signs, is not accompanied by a proportionate increase in power—a large heart with not well-filled arteries.

In support of his conclusions, Phillips gives the histories of many cases where considerable improvement ensued as the result of antisyphilitic treatment where previously cardiac tonics had failed, cases which apparently belonged to all classes recorded, both those of gummata of the heart wall and fibroid change with or without dilatation.

[1] Egger, Münch. Med. Wochenschrift, Nov. 10, 1896. [2] Campbell, Lancet, Oct. 10, 1896. [3] Satterthwaite, New York Med. Record, April 10, 1897. [4] Phear, Clinical Journal, May 12 and 19, 1897. [5] Dock, New York Med. Journal, Jan. 30, 1897. [6] Sainsbury, Lancet, Oct. 17, 1896. [7] Fox, Lancet, Feb. 20, 1897. [8] Thayer and Blumer, Johns Hopkins Hosp. Bulletin, April, 1896. [9] Phillips, Lancet, Jan. 23, 1897.

### DIPHTHERIA.

### Serum Therapy.

(*Continued from page* 304.)

The significance of mixed infection in diphtheria for various complications and for the serum treatment, is also discussed by Von Dungern,[11] who has endeavoured by a series of experimental investigations to reconcile the divergent conclusions arrived at by Funk, Roux, and Martin, and others. It having been established by Roux, and Yersin, and V. Schreider that the infection of guinea-pigs with Loeffler bacillus is rendered more severe by various cocci, particularly the streptococcus erysipelatosus, Funk sought to determine how far this depended upon increased toxin production by the diphtheria bacillus under the influence of the streptococci, and how far upon increased susceptibility of the organism to the diphtheria toxin, and from the results obtained by experiments on guinea-pigs Funk concluded—(1) that the simultaneous injection of streptococci and diphtheria bacilli stimulates the latter to increased toxin formation, but that this effect is not so considerable as hitherto supposed; (2) that the simultaneous presence of the streptococci in no way inteferes with the specific action of the diphtheria anti-toxin. Roux and Martin arrived at a result directly opposite to that obtained by Funk; this they explain as being due to the anti-toxin being unable to stimulate the cells already paralysed by the streptoccus toxins.

Now, V. Dungern constantly found it possible to isolate by means of agar-plates streptococci from the tonsillar mucus of healthy individuals; they are the most common cause of ordinary tonsillitis, and of many pseudo-diphtheritic conditions, and they are almost constantly associated with Loeffler's baccillus in true diphtheria. Consequently, the mere establishment of the presence of streptococci in the false membrane cannot have any prognostic value, since they are almost regularly met with. For reasons stated, Dungern believes that we may safely accept that those cases in which streptococci grow along with the diphtheria bacillus are of a more severe type than those in which other cocci only accompany the diphtheria bacillus. It thus follows that the growing power of streptococci upon serum affords some indication as regards their virulence.

Since ox serum as a medium is less suited to streptococci than to the diphtheria bacillus, a profuse growth of both organisms on such serum indicates not only that many very vital streptococci must have been present in the membrane used for inoculation, but also that these streptococci were suited to the nutrient conditions which obtained in the human organism. It is exactly under these conditions that this form of mixed infection becomes a real danger.

From experiments by Dungern on rabbits inoculated simultaneously with streptococci and diphtheria bacilli, it seems that the diphtheria toxins are capable of breaking through the protective barrier of leucocytes, which appear at the site of inoculation of streptococci, and thus of affording to the streptococci an easy entrance to the vascular system. Whether general infection results under these conditions appears to depend entirely upon the initial virulence of the streptococci. And also in man the possibility of a severe septicæmia, following on the mixed infection of diphtheria bacilli and streptococci entirely depends upon the virulence of the streptococci in question for man. The manner of growth on serum and the testing of the virulence on animals can afford us a certain amount of information as to such a possibility.

Finally, there still remains for consideration the influence of mixed infection with streptococci on the applicability of the specific anti-toxic serum therapeutics. Different authors again express diverse opinions. Funk, employing guinea-pigs which were only slightly susceptible to the streptococcus used, was of opinion that streptococcic mixed infection does not at all interfere with the specific treatment. Roux and Martin, on the contrary, employing rabbits, found that streptococcic mixed infection is extremely difficult to influence by anti-diphtheria serum. An explanation of these discrepancies Dungern sees in the relative virulence of the streptococci employed. He concludes that with slightly virulent streptococci, which are unable to give rise to a general septicæmia (*cf.* streptococcus I.), the anti-toxic serum will act exactly as in pure diphtheria. But with more virulent streptococci, which under the influence of diphtheria bacilli readily produce a general infection (*cf.* streptococcus II.), the diphtheria anti-toxic serum can only exert a curative influence when employed very early before the onset of septicæmia, and that later it should be used together with the anti-streptococcic serum. Of antiseptic methods little is to be expected. Hence the participation of other bacteria in the diphtheritic affection is no indication for giving up the specific treatment with

Behring's diphtheria serum and falling back upon antiseptic methods.

We may here draw attention to a practical point determined by Kossel.[12] Investigating the question of toxin formation during the early days of growth, viz., the second, fifth, and tenth, he found that the maximum toxicity of a culture is reached in five days. Further, he collected quantities of the pellicle which forms on the surface of the cultures in broth, and after washing and freeing from bouillon till no biuret reaction was given by the washings, the bacilli were killed by chloroform vapour, and extracted with weak alkaline solution for some days. From the very slight toxicity of the washing he concluded that though there is a certain amount of intra-cellular poison, the greater portion of the toxin is elaborated from the materials of the culture medium by the bacilli within the bodies of the bacilli, but is immediately afterwards secreted.

**Nephritis and Anti-toxin.**—Goodall[13] records three cases, at the Eastern Fever Hospital, of diphtheria occurring in the subjects of nephritis, and treated with anti-toxin, aged six, five, and ten years respectively. He remarks that these three cases are interesting when considered in relation to any supposed effect of anti-toxin on the kidneys. In Cases 1 and 2 the patients were actually suffering from nephritis when the serum was injected and the renal inflammation was in no way aggravated—quite the contrary, in fact, in Case 2. In Case 3 the patient had apparently recovered from the nephritis, but there was a recurrence of the latter four days after the anti-toxin rash had appeared and while it was still present. It is possible that in this case the serum caused a fresh lesion in organs just recovering from an acute inflammation. This is the only case in which he had seen nephritis follow an injection of serum (though in one case of hæmorrhagic diphtheria he had observed hæmaturia), and the symptoms lasted only for five days. But diphtheria occurring during the third or fourth week after an attack of scarlet fever is occasionally coincident with the onset of nephritis, and it is to be noted that in Case 1 a relapse of nephritis accompanied the secondary diphtheria, the symptoms being observed before the administration of anti-toxin. Indeed, now and then a case of scarlatinal nephritis relapses from no apparent cause.

In this connection we may recall Siegert's[14] clinical and experimental observations upon the effect of the subcutaneous injection of Behring's diphtheria anti-toxin on the kidney. He finds that after the injections there occurs generally a slight transitory albuminuria and albumosuria; this was found not only in patients already suffering from diphtheria, but also in healthy children in whom the anti-toxin was injected as a prophylactic measure. In animals the injection produced similar change in the urine, and also a diminution in its quantity and specific gravity. It was suggested that the albuminuria was due to the small amount of carbolic acid in the serum, but this was easily disproved. In some cases acute parenchymatous and hæmorrhagic nephritis occurred in patients treated with the serum, but there was no evidence that this occurred unless some change had taken place in the serum used. Occasionally anuria occurred after the injection, and the same phenomenon was observed in animals. If albuminuria be already present in a case of diphtheria the injection of anti-toxin generally causes the albuminuria to disappear without evil consequences. That the alterations in the urine are generally due to mere functional disturbance in the kidney seems to be shown by the fact that even with 10 c.cm. of Behring's serum no organic lesion of the kidney could be produced in rabbits. Siegert concludes that if it can be shown that anti-toxin is a specific against diphtheria the usually slight disturbance of the function of the kidney cannot be urged against its use.

**Intubation.**—Edwin Rosenthal,[15] of Philadelphia, has stated that out of 166 severe cases of diphtheria which he had treated with anti-toxin, 76 were laryngeal, and only four patients had died. In 34 intubation was required, and of these only three had died. These results should be contrasted with some in his experience before the introduction of anti-toxin, when he had lost as many as 68 out of 100 cases. Now and then we read of a case of rather sudden death which had been attributed to anti-toxin, but Rosenthal doubts this, for he has seen sudden death before the introduction of anti-toxin, simply from moving the child, or just after intubation, &c., the cause being probably heart failure. Formerly he saw no cases of recovery from diphtheria secondary to measles, whereas now, under anti-toxin treatment, death was rare also in such cases. He mentioned the occurrence of diphtheria of the nose without any membrane in the throat. He had had no bad effects from anti-toxin, but now and then had seen temporary eruption. Hillis[16] remarks that it is well known that considerable difficulty is often experienced in feeding children while the tube is in the larynx. The difficulty is commonly overcome by placing the child on the nurse's lap on its back, with the head hanging down over the edge of the lap. Personally, he preferred to have the little patient lie on the stomach, face down, as this gave greater command over the constrictors. The difficulties encountered in removing the tube by those unaccustomed to intubation are often grave obstacles to the adoption of the method. Bayeux,[17] of Paris, has had tubes made like O'Dwyer's, without the sub-ventricular part being so long. He found that this shortened tube could be ejected from the larynx like a "stone from a cherry," by gently compressing the trachea just beneath the cricord. This form of tube seems to be used a good deal in Paris.

Louis Fischer[18] has introduced corrugated tubes made of vulcanised rubber, which *in situ* extend from the false cords to about one half to three-fourths of an inch above the bifurcation of the trachea (verified on the cadaver). The tube bulges in the centre to give it weight and to render it more self-retaining. These tubes are cheap, and the secretions do not adhere to them. Fischer has also devised a simple introducer which does away with the obturator (which blocks the lumen till removed). From numerous anatomical mensurations he proves that the narrowed part of the larynx is exactly on a level with the cricord cartilage. Every tube, the swollen part of which is not inserted beyond the cricoid, will be easily expelled. The vocal bands do not keep the tube *in situ*; it is the stenosed part of the cricoid ring. The tube must be constructed for a convenient adaptation.

[11] Ziegler's Beitrage, Bd. xxi., 1887; F. C. Moore, abstract, Med. Chron., April, 1897. [12] Cent. fur Bakt, &c., 1896, xix., p. 977; Epit. Brit. Med. Journ., Dec. 19, 1896. [13] Lancet, Nov. 21, 1896. [14] Virchow's Archiv., Nov., 1896; Brit. Med. Journ., Dec. 18, 1896. [15] New York Med. Rec., Jan. 2, 1897. [16] New York Med. Rec., Nov. 28, 1896. [17] Presse Med., Jan. 20, 1897. [18] Med. Rec., June 19.

### DISEASES OF CHILDREN.

(*Continued from page* 305.)

**Idiopathic Osteopsathyrosis (Fragilitas Ossium).**—An interesting account of this condition, as affecting infants and children, has been given by Dr. Crozer Griffiths.[9] The symptomatic osteopsathyrosis may be dependent on rickets, or osteomalacia, or scurvy, or syphilis, although the latter two are more frequently associated with separation of the epiphyses. In other cases—to which his paper is directed—no such cause can be discovered. He describes one case, that of an infant in whom the femora were found to be broken two days after birth. Other fractures followed, until, before the age of three years, the child had had some 17 or 18 in all, the femora and ribs being most frequently affected. The literature of the subject is reviewed, and 57 collected cases are summarised by the author. These he groups into three classes: (*a*) Those showing direct inheritance, (*b*) those showing a family predisposition, and (*c*) isolated cases. As regards etiology, inheritance through the male line was present in a considerable proportion of cases, but nothing else seems to have been determined. He thinks that syphilis may be dismissed; and regarding rickets, he holds that no definite association has been proved, the special tendency to fracture appearing often at an age before rickets has developed. An interesting feature of the affection is that in nearly all the cases union of the broken bones was prompt. The position of the fracture is entirely independent of the relation of diaphysis and epiphysis, and in very few instances is there even the supposition that the condition might consist in the separation of the epiphysis. When refracture occurred, it might be at the seat of a former fracture, but was more commonly away from it. The prognosis is, as a rule, unfavourable; for when this tendency to fracture is once established, it is apt to persist in spite of treatment. The condition does not seem to affect the general health or the prospect of life.

**Lumbar Puncture.**—From a study of twenty-nine cases in which this method of diagnosis was employed, Dr. Wentworth[10] draws the following conclusions. In cases of meningitis the cerebro-spinal fluid is invariably cloudy when withdrawn, the cloudiness being caused by the presence of cells, and a variable amount of fibrin is present. These points serve to determine the presence of meningitis, as the normal cerebro-spinal fluid is perfectly clear, and contains neither cells nor fibrin. Further, while under normal conditions this fluid contains a faint trace of albumin, in meningitis the amount is always increased.

**Congenital Gastric Spasm.**—This condition, which is also described under the title Congenital Hypertrophy and Stenosis of the Pylorus, is fully discussed by Dr. John Thomson.[11] He relates a case that came under his own observation, and has collected fifteen other published cases. The leading symptom is vomiting, which occurs at first at intervals, but as the case proceeds comes to follow every attempt to swallow fluid. No medicinal or other treatment has been found to prevent the vomiting. On examination of the abdomen it is possible in some cases to perceive peristaltic movements of the stomach and to palpate the hard hypertrophied pylorus. The duration of life varies from three weeks to a little over six months. The author thinks that the essential lesion is not a muscular but a nervous one, a functional disorder of the nerves of the stomach and pylorus leading to ill co-ordinated and therefore antagonistic action of their muscular arrangements.

**Late Rickets.**—Dr. Alexander James[12] has recorded a case of very late rickets, the onset of illness being at the seventeenth year. The affection commenced with shooting pains in the thighs, which later became bent. He was first seen when twenty years old, and was then an undersized lad with a large head, a typically rickety chest, and bony enlargements, specially marked at the wrists and knee joints. The case is fully described, and the paper is illustrated by excellent photographs and skiagraphs.

**Neurotic Symptoms of Uricacidæmia.**—In the case of a child who partook largely of butchers' meat, Dr. David Drummond[13] found that a series of nervous symptoms disappeared when he was put on a milk diet, and returned when meat was again given freely. He regards this condition as one of uricacidæmia, and in similar cases has always found an excessive amount of uric acid in the urine. The symptoms are insidious in their origin, and develop slowly. Headache, loss of appetite, constipation, anæmia, loss of flesh, subnormal temperature, slow and tense pulse, cold hands and feet, mental lethargy, and occasional attacks of giddiness are the chief symptoms. In the majority of cases the substitution of a natural diet will effect a speedy cure.

**Chicken Pox.**—Dr. J. J. Walsh,[14] from his experience gained in an epidemic of this affection, is of opinion that it is neither to be ignored as a trifling ailment, nor differentiated from small-pox without considerable difficulty in many cases. In opposition to the teaching of many authors, he has found that there may be high fever (104° Fahr.), a macular and papular rash, and an inflammatory areola around the vesicles, which may be confluent, umbilicated, partitioned, and pustular, and finally may leave depressed cicatrices not unlike "pock marks." He recommends that great care should be taken to prevent the occurrence of nephritis, and that the vesicles be washed with a 5 per cent. solution of carbolic acid, so as to prevent pustulation and scarring.

9 Am. Jour. Med. Sci., April, 1897. 10 Bost. Med. and Surg. Jour., Aug. 6, 1896, and Am. Jour. Med. Sci., Jan., 1897. 11 The Scot. Med. and Surg. Jour., June, 1897. 12 Ibid., Jan., 1897. 13 The Lancet, May 15, 1897. 14 The Therap. Gazette, Oct. 15, 1896.

